# Respiratory viral infections in hospitalized adults: a comparative clinico-laboratory study of RSV, HMPV, and influenza

**DOI:** 10.1186/s12985-025-02924-2

**Published:** 2025-08-28

**Authors:** Santhosha Devadiga, Prasad Varamballi, Ujwal Shetty, Chiranjay Mukhopadhyay, Anup Jayaram

**Affiliations:** Manipal Institute of Virology, Manipal Academy of Higher Education, Madhava Nagar, Manipal, 576104 Karnataka India

**Keywords:** Human respiratory syncytial virus, Human metapneumovirus, Influenza, Clinical, Laboratory, Comparative study

## Abstract

**Background:**

Respiratory viral infections, including Human Metapneumovirus (HMPV), Influenza (Flu), and human Respiratory Syncytial Virus (hRSV), are major global health concerns. While their impact on vulnerable groups is known, their characteristics in healthy adults (18–65 years) are less clear. This study aimed to determine the incidence and clinical-laboratory features of RSV and HMPV in this population and compared them with those of Influenza A(H1N1) and influenza A(H3N2) for improved epidemiological and diagnostic understanding.

**Methodology:**

A retrospective analysis was conducted on data from an Acute Febrile Illness surveillance (2016–2018) in Manipal, India. The study included 96 HMPV, 68 hRSV, 75 Influenza A(H1N1), and 76 Influenza A(H3N2) positive hospitalized adults with fever (≥ 38 °C) and respiratory illness confirmed by RT‒PCR. Clinical and laboratory data collected within the first 8 days of illness were statistically analyzed.

**Results:**

The annual incidence rates of hRSV (0.33%-1.59%) and HMPV (0.14%-1.79%) varied. Coryza was common, but cough was most frequent in HMPV (97.9%). HMPV also resulted in increased rates of shortness of breath and chest pain. Leucopenia was most common in Influenza A(H1N1) patients, and thrombocytopenia was most common in hRSV patients. Significantly elevated leukocyte and platelet counts were observed in HMPV patients. Liver enzyme abnormalities are relatively common in hRSV and Influenza A(H1N1) patients. Symptom progression and laboratory trends revealed distinct patterns across the viruses.

**Conclusion:**

Despite overlapping initial symptoms, HMPV, hRSV, and influenza resulted in different clinical and laboratory profiles in adults. HMPV was associated with more prominent lower respiratory symptoms and a stronger inflammatory response. These distinctions can aid in the clinical differentiation and management of these common respiratory viruses in adults, highlighting the importance of timely diagnosis for improved patient care and public health strategies.

## Graphical Abstract

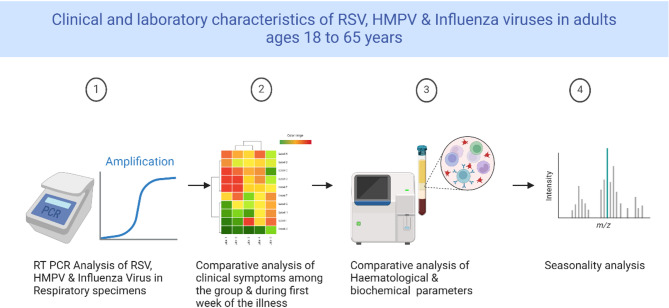

## Introduction

Respiratory viral infections are a significant global health concern, affecting millions of people annually and contributing to substantial morbidity and healthcare burdens. Human Metapneumovirus (HMPV), Influenza (Flu), and human Respiratory Syncytial Virus (hRSV) are the leading viral pathogens responsible for acute respiratory tract infections across different age groups [1]. These infections present with a wide spectrum of clinical manifestations, ranging from mild upper respiratory symptoms to severe pneumonia, acute respiratory distress syndrome (ARDS), and even mortality, particularly in vulnerable populations such as elderly and immunocompromised individuals [2]. Despite the seasonal and pandemic nature of these infections, their impact on otherwise healthy adults aged 18–65 years remains underexplored.

Understanding the incidence, clinical presentations, and laboratory findings of respiratory viral infections in adults is essential for understanding the nature of infection and circulation patterns, improving diagnostic algorithms and guiding therapeutic interventions. Clinical-laboratory features such as leukopenia, thrombocytopenia, elevated inflammatory markers (C-reactive protein, erythrocyte sedimentation rate), and liver enzyme abnormalities can aid in differentiating viral etiologies and predicting disease severity [3]. Moreover, variations in symptoms such as fever duration, respiratory distress, and the systemic inflammatory response among different viral pathogens can provide insights into disease progression and management strategies.

Given the emerging threats of seasonal and novel respiratory viruses, including the potential for coinfections and secondary complications, continuous surveillance and characterization of their clinical and laboratory profiles are crucial [4, 5]. This study aimed to assess the incidence and clinical-laboratory characteristics of influenza, hRSV and HMPV infections in adults aged 18–65 years, providing valuable epidemiological and diagnostic insights. These data can support evidence-based clinical decision-making and inform public health strategies to mitigate the impact of respiratory viral infections.

## Methodology

Study design: A retrospective observational study was conducted to analyze clinical data from patients diagnosed with HMPV, hRSV and Influenza. A total of 68 hRSV and 96 HMPV-positive patients were included in the study. Respiratory specimens collected as part of Acute Febrile Illness (AFI) surveillance study conducted by Manipal Institute of Virology (MIV), with a case definition of patients admitted to the hospital with fever (≥ 38 °C) and acute respiratory illness, were included, and samples were tested for various viral pathogens, including hRSV, HMPV and Influenza virus, via molecular and serological methods from 2016 to 2018 [6].

hRSV- and HMPV-positive individuals were defined as those individuals whose results were positive according to reverse transcription‒polymerase chain reaction (RT‒PCR) analysis of respiratory samples (i.e., nasal/nasopharyngeal swabs). Briefly, total nucleic acid was isolated from nasal/throat swabs in viral transport medium (VTM) via a QIAmp viral RNA Mini Kit (#52906, Qiagen, Hilden, Germany) according to the manufacturer’s instructions. Multiplex real-time RT‒PCR was performed with primers and probes targeting HMPV and hRSV via a Respiratory Pathogens 21 Kit (#11373972, Fast Track Diagnostics - FTD, Luxembourg). RT‒PCR was performed via a AgPath-ID™ One-step RT‒PCR Kit (#4387391, Applied Biosystems, Foster City, USA). The reaction was performed in a QuantStudio™ 5 PCR system (#A28569, Applied Biosystems, Foster City, USA). The results are expressed as cycle threshold (Ct) values. Laboratory-confirmed cases were classified as upper respiratory tract illness (URTI) or lower respiratory tract illness (LRTI) on the basis of clinical symptoms defined by the World Health Organization (WHO) [7]. Age group matched 75 Influenza A(H1N1) positive, and 76 Influenza A(H3N2) positive cases were included for the comparison by random sampling method. Demographic, clinical and laboratory data of hRSV, HMPV and Influenza A positive patients were obtained from the AFI study database after ethical clearance was obtained and used for analysis. The current study was reviewed and approved by the Institutional Ethical Committee, Manipal Academy of Higher Education (IEC No: UEC/32/2013-14, MUEC/Renewal-08/2017, MIV/AFI-IEC/2023).

### Data analysis

We assessed clinical and laboratory features during the first 8 days of post onset of illness. Upper, lower respiratory and gastrointestinal symptoms between the group and during the course of illness were analyzed. Hematological and biochemical parameters of the hRSV, HMPV and Influenza A virus positive cases were obtained from the AFI study database and analyzed using EZR version 1.68 (Easy R) [8]. For analysis of continuous variables, one-way ANOVA test and analysis of categorical variables, the chi-square test was used. A *p*-value of < 0.05 was set as the level of statistical significance.

## Results

The annual incidences of hRSV for 2016, 2017 and 2018 were 1.19% (34/2855), 0.33% (16/4847) and 1.59% (18/1126), respectively, and those of HMPV were 0.14% (4/2855), 1.79% (87/4847), and 0.44% (5/1126), respectively. The frequency of symptoms in patients infected with human Respiratory Syncytial Virus (hRSV), Human Metapneumovirus (HMPV), Influenza A (H1N1) and Influenza A(H3N2) are categorized into upper respiratory tract infections (URTIs), lower respiratory tract infections (LRTIs), and gastrointestinal symptoms. Coryza (runny nose) was most common in HMPV (85.4%), followed by Influenza A(H3N2) (72.3%), Influenza A(H1N1) (74.6%), and hRSV (70.6%). Sore throat was reported more frequently in Influenza A(H1N1) (56%) and Influenza A(H3N2) (55.2%) patients than in hRSV (50%) patients. LRTI symptoms such as cough were common across all viruses, with the highest frequencies of HMPV (97.9%) and Influenza A(H1N1) (97.3%). Shortness of breath was most reported in HMPV (22.9%), whereas hRSV, Influenza A(H1N1), and Influenza A(H3N2) showed similar frequencies (16–19%). The rate of chest pain was highest for HMPV (23.9%), followed by Influenza A(H3N2) (22.3%), while Influenza A(H1N1) (10.6%) had the lowest rate. Gastrointestinal symptoms such as nausea and vomiting were most common in Influenza A(H3N2) patients (46.1% and 67.1%, respectively), followed by Influenza A(H1N1) patients (37.3% and 32%). Chills and myalgia (muscle aches), headache and weakness were very common across all infections, ranging from 84.2 to 91.6% (Table 1).

**Table 1:** Frequency of clinical symptoms in patients infected with human respiratory syncytial virus (hRSV), human metapneumovirus (HMPV), influenza A(H1N1) and influenza A(H3N2)

Variables	hRSV *N* (%) (*n* = 68)	HMPV *N* (%) (*n* = 96)	Influenza A	
			H1N1 N (%) (*n* = 75)	H3N2 N (%) (*n* = 76)
**URTI symptoms**				
Coryza	48(70.6)	82(85.4)	56(74.6)	55(72.3)
Sore throat/pharyngitis	34(50)	51(53.1)	42(56)	42(55.2)
**LRTI symptoms**				
Cough	63(92.6)	94(97.9)	73(97.3)	70(92.1)
Shortness of breath	13(19.1)	22(22.9)	12(16)	13(17.1)
Chest pain	14(20.6)	23(23.9)	8(10.6)	17(22.3)
**Gastrointestinal symptoms**				
Nausea	20(29.4)	32(33.3)	28(37.3)	35(46.1)
Vomiting	14(20.6)	19(19.8)	24(32)	51(67.1)
Abdominal pain	13(19.1)	23(23.9)	20(26.6)	15(19.7)
**Other symptoms**				
Chills	44(64.7)	78(81.2)	68(90.6)	66(86.8)
Myalgia/muscle ache	54(79.4)	77(80.2)	71(94.6)	64(84.2)
Arthralgia/joint pains	36(52.9)	58(60.4)	48(64)	49(64.4)
Headache	59(86.7)	88(91.6)	65(86.6)	64(84.2)
weakness	63(92.6)	84(87.5)	67(89.3)	67(88.1)
Conjunctivitis congestion	8(11.7)	3(3.1)	2(2.6)	4(5.2)

**Fig. 1:**
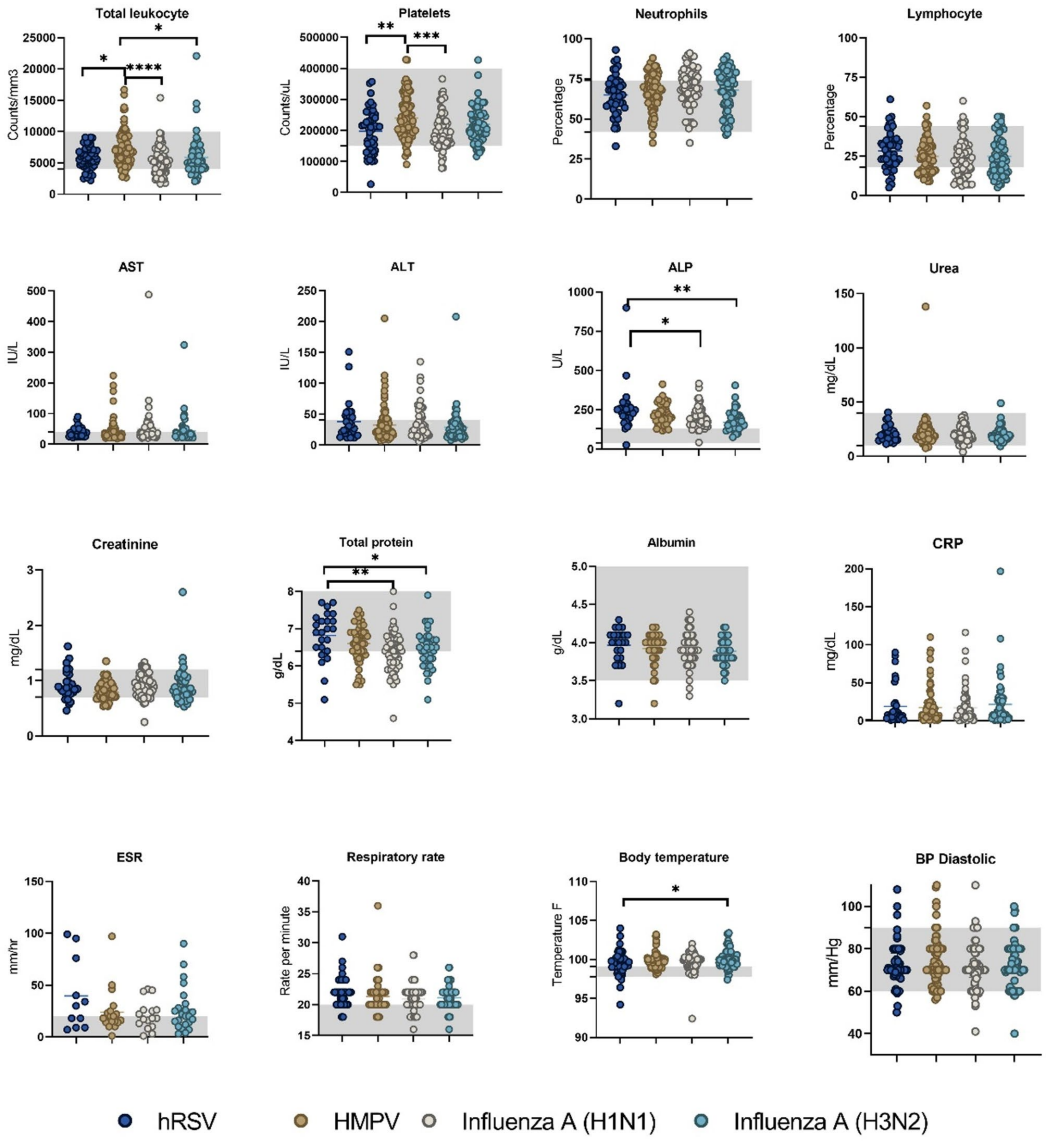
Comparative analysis of hematological and biochemical parameters in patients with hRSV, HMPV, Influenza A (H1N1), and Influenza A (H3N2) infections. The line represents the median; the gray band in the graphs indicates the normal range. Statistical significance (*p* value and 95% CI) was determined by one-way ANOVA followed by Tukey’s multiple comparisons test. Graphs were generated via GraphPad Prism version 8.4.2

The mean age of the hRSV patients was 40.26 years, and that of the HMPV patients was 34.4 years. Leucopenia was most frequently observed in Influenza A(H1N1) patients (31.8%), whereas thrombocytopenia was most common in hRSV patients (28%). Neutrophilia (> 70%) was more common in Influenza A(H1N1) (52.1%) and HMPV (41.7%) patients. Liver function abnormalities, including elevated aspartate transaminase (AST) and alanine transaminase (ALT) levels, were most prevalent in hRSV (44.1% and 35.3%, respectively) and Influenza A(H1N1) (43.9% and 28.7%) patients. Alkaline phosphatase levels were frequently elevated across all groups. Inflammatory markers, such as C-reactive protein (CRP), were elevated in nearly half of the patients across all infections (Table 2). Notably, the total leukocyte count, platelet count, alkaline phosphatase (ALP) level and total protein level significantly differed among the hRSV and HMPV groups. The platelet count is significantly greater in HMPV compared to Influenza A(H1N1) and hRSV (Fig. 1). The total leukocyte count is also significantly greater in HMPV compared to Influenza A(H1N1), hRSV and Influenza A(H3N2). ALP levels are lower in hRSV than in the other groups, and total protein levels are significantly lower in Influenza A(H1N1) patients. Additionally, body temperature is significantly elevated in individuals with Influenza A(H1N1). Other parameters, such as the CRP level, erythrocyte sedimentation rate (ESR), respiratory rate, and diastolic blood pressure, did not significantly differ among the groups.

**Table 2:** Laboratory characteristics of patients hospitalized with hRSV, HMPV, influenza A (H1N1) and influenza A(H3N2)

Variables	hRSV (*n* = 68)	HMPV (*n* = 96)	Influenza A	
			H1N1 (*n* = 75)	H3N2 (*n* = 76)
Mean Age in years (SD)	40.26(14.4)	34.4(11.6)	37.2(11.9)	35.1(13.6)
Mean duration of fever before hospitalization (days)	4.4 (1.9)	4.6(2.06)	3.58(1.4)	3.6(1.7)
Body temperature > 99 °F	49(74.2%)	73(77.6%)	53(80.3%)	63(86.3%)
Hypotension blood pressure < 60/90 mmHg	3(4.6%)	3(3.4%)	5(6.9%)	3(4.2%)
Respiratory rate > 24/minute	15(22.7%)	4(4.2%)	3(4.1%)	2(2.6%)
Leukopenia < 4000 cells/mm3	7(13.7%)	7(7.6%)	22(31.8%)	13(18.3%)
Thrombocytopenia < 150 × 10^3^/ul	14(28%)	6(6.5%)	10(14.9%)	7(9.8%)
Neutrophil percentage > 70%	13(26.5%)	38(41.7%)	36(52.1%)	35(47.9%)
Lymphocyte percentage > 40%	8(16.3%)	8(8.7%)	8(11.5%)	12(16.4%)
Aspartate aminotransferase > 40 IU/L	15(44.1%)	27(30.6%)	29(43.9%)	16(27.6%)
Alanine aminotransferase > 40 IU/L	12(35.3%)	21(23.8%)	19(28.7%)	9(15.5%)
Alkaline phosphatase > 140 IU/L	19(73%)	42(89.3%)	48(78.6%)	27(69.2%)
Erythrocyte Sedimentation Rate > 20 mm/h	6(54.5%)	7(35%)	6(35.3%)	11(44%)
Urea levels > 40 mg/dl	0(0)	1(1.4%)	0(0)	1(1.9%)
Creatinine levels > 1.2 mg/dl	0(0)	1(1.5%)	1(1.5%)	3(6.5%)
C-Reactive protein levels > 10 mg/dl	14(42.4%)	39(46.4%)	32(49.2%)	26(48.1%)

**Fig. 2:**
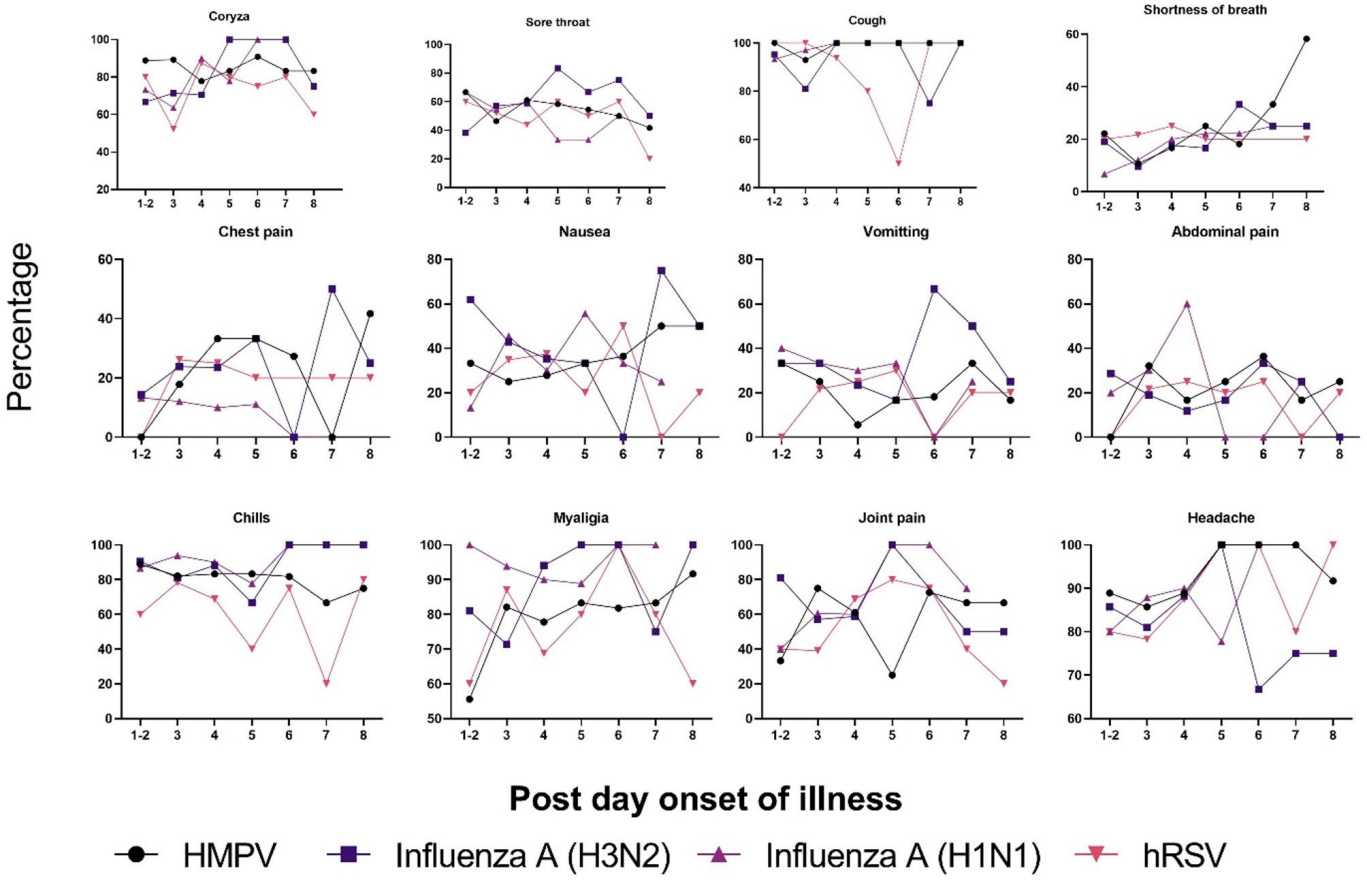
Temporal progression of various symptoms in patients infected with HMPV, Influenza A (H1N1), Influenza A (H3N2) and hRSV over the course of illness

Coryza and sore throat are consistently highly prevalent across all infections, particularly in the early days. Shortness of breath is initially low but increases notably in HMPV cases by days 6–8. Chest pain, nausea, vomiting, and abdominal pain exhibit fluctuating patterns, with some spikes observed later in the illness. Chills and myalgia remain prevalent throughout, especially in Influenza A(H3N2) and Influenza A(H1N1) cases, with myalgia showing the highest overall consistency. Joint pain and headache also followed variable trends, but headache remained notably high in all groups. Overall, while some symptoms, such as cough, chills, and myalgia, are common and persistent, others, such as shortness of breath and gastrointestinal symptoms, are more variable, potentially indicating differing disease severity and symptom progression among viral infections (Fig. 2).

**Fig. 3:**
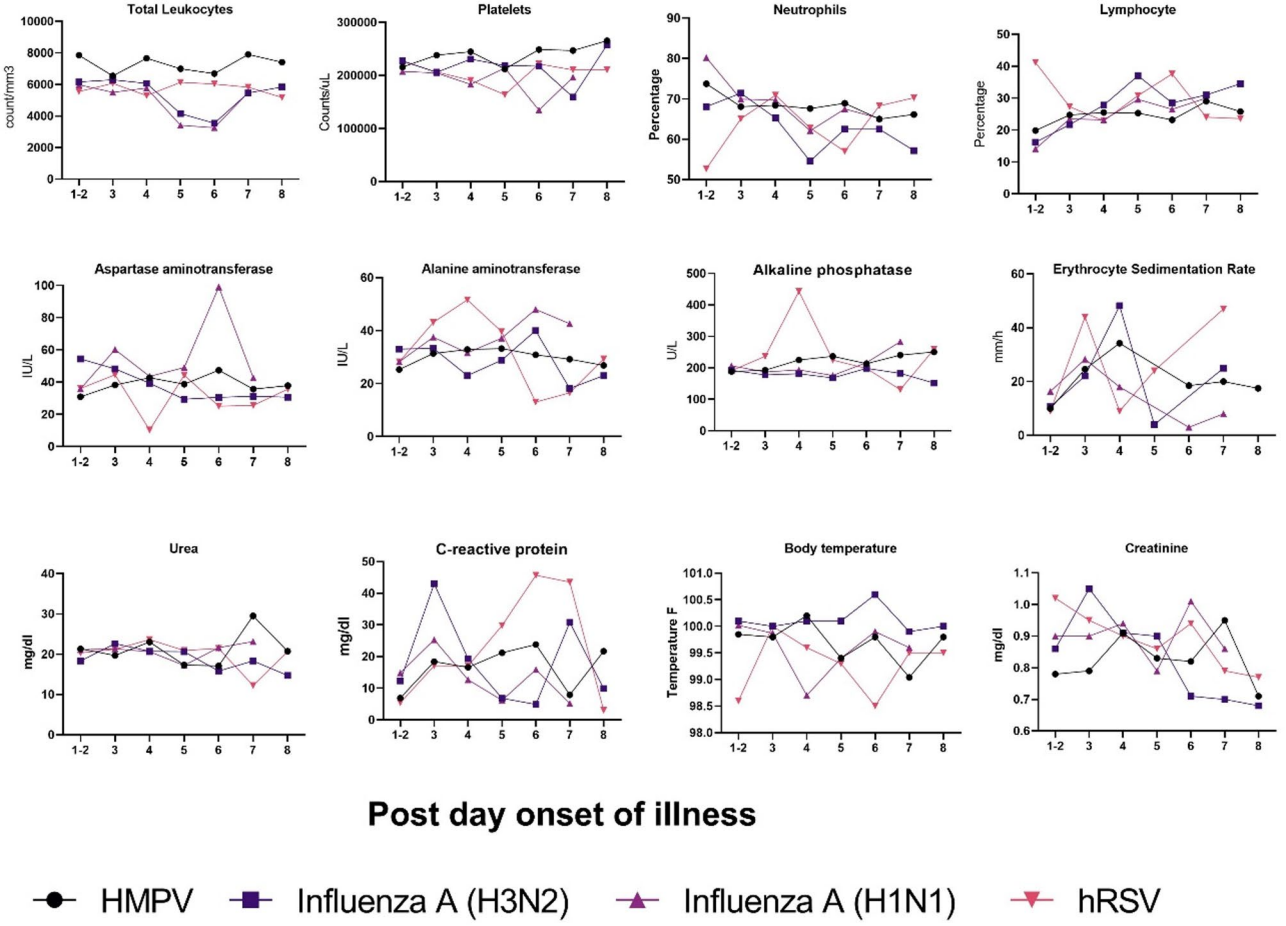
Temporal trends in various laboratory parameters in patients infected with HMPV, Influenza A (H3N2), Influenza A (H1N1), or hRSV over the course of illness

The total leukocyte count remains relatively stable, with a slight decline in some cases, whereas the neutrophil percentage decreases over time, particularly in hRSV cases, with a corresponding increase in the lymphocyte percentage. Platelet levels fluctuate, with noticeable dips in hRSV and Influenza A(H1N1) cases. Liver function markers, including AST and ALT, show intermittent peaks, particularly in hRSV and Influenza A(H1N1) infections, whereas ALP levels exhibit variable spikes. Inflammatory markers such as ESR and CRP fluctuate, with CRP peaking prominently for around 3–5 days in hRSV and HMPV patients. Urea and creatinine levels remain relatively stable, with slight elevations in later illness stages, particularly in HMPV and Influenza A(H3N2) patients. Body temperature remains at approximately 99–100 °F across all infections, with minor fluctuations (Fig. 3).

## Discussion

Human Respiratory syncytial virus (hRSV) and human metapneumovirus (HMPV) are significant viral pathogens that cause acute respiratory infections (ARIs) in children and elderly adults [9, 10]. While hRSV has been extensively studied in infants and elderly individuals, its impact on otherwise healthy adults remains underappreciated. HMPV, a paramyxovirus closely related to hRSV, has also been identified as an important contributor to respiratory disease in adults. Studies have shown that the annual incidence of hRSV in adults ranges between 2% and 7%, whereas HMPV infections are observed in 1–5% of cases [3, 10].

Clinically, hRSV, HMPV and influenza present with overlapping symptoms, including cough, fever, coryza, and gastrointestinal involvement. Lower respiratory tract symptoms such as cough and shortness of breath are frequently observed across infections, with HMPV showing the highest rates of cough (97.9%), corroborating previous reports on its significant involvement in bronchiolitis and pneumonia. However, hRSV tends to cause more severe lower respiratory tract involvement, particularly in individuals with comorbidities such as asthma, chronic obstructive pulmonary disease (COPD), and cardiovascular disease [11]. HMPV infections, while similar in presentation, are more frequently associated with bronchiolitis-like illness in adults. Hospitalized Influenza A(H1N1) patients exhibit leukopenia (31.8%), with thrombocytopenia being most common in hRSV patients (28%). Liver function abnormalities, particularly elevated AST and ALT levels in hRSV and Influenza A(H1N1) patients, are in agreement with prior studies showing hepatic involvement in severe viral respiratory infections. Elevated inflammatory markers, such as C-reactive protein (CRP) and the erythrocyte sedimentation rate (ESR), along with liver function abnormalities, have been reported in some studies, particularly in patients with severe infections [12]. Additionally, hypoxia and hypotension are observed in severe hRSV patients requiring intensive care. The findings of this study highlight distinct hematological and biochemical profiles associated with hRSV, HMPV, Influenza A(H1N1) and Influenza A(H3N2) infections. Elevated leukocyte and platelet counts in HMPV suggest a stronger inflammatory response, whereas lower total protein levels in Influenza A(H1N1) may indicate disease severity. Significant variations in ALP further distinguish these infections. These differences could aid in the clinical differentiation and management of viral respiratory infections.

Symptom progression and laboratory trends provide further insight into disease severity and trajectory across infections. The increase in shortness of breath in HMPV cases from days 6–8 parallels the literature highlighting prolonged lower respiratory involvement in HMPV than in hRSV and influenza. The persistence of chills, myalgia, and headache across all infections, particularly in influenza cases, aligns with reports that systemic symptoms are more pronounced in influenza patients because of their greater cytokine response [13]. Additionally, fluctuating platelet levels in hRSV and Influenza A(H1N1), along with intermittent peaks in AST and ALT, mirror previously documented cases where these infections result in transient hematological and hepatic dysfunction. The relatively stable renal function markers with minor late-stage elevations in HMPV and Influenza A(H3N2) are consistent with reports indicating minimal direct renal involvement in these infections.

The laboratory diagnosis of hRSV and HMPV is achieved primarily through molecular techniques such as real-time polymerase chain reaction (RT‒PCR), which is more sensitive than traditional viral culture or antigen detection methods. Despite the similarities in clinical presentation, differences in inflammatory responses and organ involvement may aid in distinguishing between hRSV and HMPV. Current treatment strategies remain supportive, with oxygen therapy, bronchodilators, and corticosteroids being used in severe cases, although antiviral treatments such as hRSV-targeted monoclonal antibodies are under investigation. Given the burden of hRSV and HMPV in adults, particularly those with underlying health conditions, increased surveillance and the development of targeted vaccines and therapeutics are crucial for mitigating their impact [14].

This study presents a comprehensive comparison of the clinical symptoms, demographic characteristics, laboratory findings, and temporal trends associated with hRSV, HMPV, Influenza A(H1N1), and Influenza A(H3N2) infections in adults over multiple years. While most existing studies on respiratory viral infections have focused on children or elderly individuals, detailed incidence rates of hRSV and HMPV in adults have rarely been reported. The observed seasonal variation in incidence, such as the increase in HMPV from 0.14 to 1.79%, is not well described and is a notable epidemiological finding. Furthermore, this study provides dynamic insights into disease progression and the kinetics of both clinical symptoms and laboratory parameters.

This study has several limitations: the sample size and assessment of symptoms and laboratory markers of disease progression were relatively limited and confined to the first 8 days of illness, and the data were derived on the basis of onset of symptoms and the day of admission. This may not fully capture the clinical course, especially in patients with prolonged or biphasic symptoms. Furthermore, coinfections with bacterial pathogens were not evaluated, which could have influenced both the clinical presentation and laboratory findings.

## Conclusion

Overall, hRSV, HMPV, and influenza A subtypes H1N1 and H3N2 share overlapping clinical features, and their progression, severity, and systemic impact differ, potentially guiding targeted clinical management strategies. Highlights the increased awareness among medical practitioners of this disease, and timely testing and diagnosis can lead to better patient management and improved public health interventions. Increased surveillance and the development of targeted vaccines and therapeutics are crucial for mitigating their impact.

This study highlights the necessity for virus-specific diagnostic, therapeutic, and surveillance strategies in managing acute viral respiratory infections. These findings offer valuable clinical and public health insights into the distinct symptomatology, laboratory profiles, and disease progression associated with hRSV, HMPV, and influenza A subtypes H1N1 and H3N2. The consistently high prevalence of coryza and cough across all infections underscores their utility as initial screening symptoms. However, the presence of specific features such as chest pain and dyspnea in HMPV and gastrointestinal manifestations in influenza A infections can aid in early differential diagnosis. Laboratory abnormalities, including AST, ALT, total leukocyte count, and platelet counts in hRSV, HMPV, and influenza cases, suggest hepatic involvement, supporting the routine inclusion of liver function tests in the evaluation of febrile respiratory illness. Temporal shifts in lymphocyte and neutrophil counts, particularly in hRSV, may reflect stages of viral clearance or the onset of secondary bacterial infections, thereby informing antimicrobial stewardship practices. Finally, the substantial interannual variability in the incidence of hRSV and HMPV underscores the importance of sustained, adaptive surveillance systems to monitor epidemiologic trends and inform public health interventions.

This study provides valuable insights into the incidence and clinical-laboratory characteristics of influenza, hRSV, and HMPV infections in adults aged 18–65 years. While these viruses share overlapping initial symptoms, our findings highlight distinct patterns in their clinical presentation, laboratory profiles, and disease progression. Notably, HMPV was associated with a greater frequency of lower respiratory tract symptoms and a more pronounced inflammatory response than were hRSV and influenza. Furthermore, specific hematological and biochemical markers, such as elevated leukocyte and platelet counts in HMPV and liver enzyme abnormalities in hRSV and influenza, may aid in clinical differentiation. These findings underscore the importance of considering these distinctions for improved diagnostic algorithms, targeted clinical management strategies, and enhanced public health surveillance to mitigate the impact of these common respiratory viral infections in the adult population.

## Data Availability

No datasets were generated or analysed during the current study.
